# Low Connexin Channel-Dependent Intercellular Communication in Human Adult Hematopoietic Progenitor/Stem Cells: Probing Mechanisms of Autologous Stem Cell Therapy

**DOI:** 10.3109/15419061003653763

**Published:** 2010-03-19

**Authors:** Jian Yang, Richard L Darley, Maurice Hallett, W Howard Evans

**Affiliations:** 1 Department of Medical Biochemistry and Immunology, Cardiff University Medical School, Cardiff, Wales, UK; 2 Department of Wales Heart Research Institute, Cardiff University Medical School, Cardiff, Wales, UK; 3 Department of Haematology, Cardiff University Medical School, Cardiff, Wales, UK; 4 Department of Surgery, Cardiff University Medical School, Cardiff, Wales, UK

**Keywords:** adult human bone marrow, cardiac repair, CD34^+^, cell adhesion molecules, cord blood

## Abstract

Human bone marrow is a clinical source of autologous progenitor stem cells showing promise for cardiac repair following ischemic insult. Functional improvements following delivery of adult bone marrow CD34^+^ cells into heart tissue may require metabolic/electrical communication between participating cells. Since connexin43 (Cx43) channels are implicated in cardiogenesis and provide intercellular connectivity in the heart, the authors analyzed the expression of 20 connexins (Cx) in CD34^+^ cells and in monocytes and granulocytes in bone marrow and spinal cord. Reverse transcriptase-polymerase chain reaction (RT-PCR) detected only low expression of Cx43 and Cx37. Very low level dye coupling was detected by flow cytometry between CD34^+^ cells and other Cx43 expressing cells, including HL-1 cardiac cells, and was not inhibited by specific gap junction inhibitors. The results indicate that CD34^+^ cells are unlikely to communicate via gap junctions and the authors conclude that use of CD34^+^ cells to repair damaged hearts is unlikely to involve gap junctions. The results concur with the hypothesis that bone marrow cells elicit improved cardiac function through release of undefined paracrine mediators.

## INTRODUCTION

Human adult progenitor stem cells exhibiting pluripotent properties are a potential therapy for inducing cardiac repair following damage caused by ischemia (Martin-Rendon et al. 2008; Murry et al. 2005; Psaltis et al. 2008; Segers and Lee 2008; Yip et al. 2008). The clinical outcomes of injecting/perfusing autologous adult bone marrow stem cells into heart muscle or into coronary blood vessels have been variable and controversial and the underlying mechanisms by which bone marrow stem cells can repair heart tissue are unclear (Balsam et al. 2004; Passier et al. 2008; Roell et al. 2007).

One mechanism by which progenitor bone marrow (BM)-derived stem cells, defined by CD34 antigen expression (Sitnicka et al. 2003; Zhang et al. 2007), improve heart function could involve their participation in direct local intercellular signaling. Cell interactions are complex and include intercellular signaling across gap junctions as well as paracrine signaling that may implicate the component connexin hemichannels between stem cells and cardiac cells. We examined therefore whether connexin proteins that provide membrane-traversing channels underpinning functional coupling are present in bone marrow cells administered in cardiac repair therapy. Connexins are also involved in cell adhesion processes (Cotrina et al. 2008) and their importance, together with other proteins, e.g., cadherins (Zhang et al. 1998) in cell adhesion and cohesion, has long been appreciated. Encouraging these studies is the fact that cardiac tissue is a rich depository of connexin (Cx)43, a protein that plays important roles in human cardiogenesis (Moore et al. 2008) and is a determinant of myocardial infarct size (Kanno et al. 2003).

To determine whether progenitor stem cells, especially CD34^+^ cells, or myeloid subpopulations purified from BM or cord blood (CB) express connexins and engage in communication with resident cardiac cells via membrane channels, we carried out a comprehensive study of connexin expression in adult BM and CB cells. We also investigated their intercellular coupling competence using dye transfer approaches. Connexins are a 22 member highly conserved family of proteins in humans (Willecke et al. 2002). They oligomerize in the endoplasmic reticu-lum into hemichannels that are delivered to the plasma membrane where they dock with partner hemichannels exposed on closely aligned neighboring cells and generate gap junction intercellular channels (Evans et al. 2006). Flow-cytometric studies using specific connexin channel inhibitors were carried out to establish whether any intercellular coupling detected was mediated by gap junctions. The results show that adult BM and CB progenitor stem cells have a very low capacity to communicate in a connexin-dependent manner and support an emerging view that autologous bone marrow cells may contribute to improved cardiac output following a cardiac infarction not by direct intercellular communication but rather by other mechanisms that may include secretion of growth factors and cytokines that promote proangiogenic effects.

## METHODS

RNA expression of 20 human connexins and N-cadherin was carried out using standard reverse transcriptase-polymerase chain reaction (RT-PCR) techniques as previously described (Oviedo-Orta et al. 2000). The primers to each of the Cxs used are listed in Table I. Briefly, total RNA was extracted using the RNeasy mini kit (Qiagen, Germany) and contaminating genomic DNA removed by treatment with RNase-free DNase I (Qiagen). First-strand cDNA was synthesized with 1 μg total RNA using the protoscriptII RT-PCR kit (New England Biolabs). Since most connexins are encoded within a single exon, a reverse transcriptase-free reaction was performed to demonstrate absence of genomic DNA contamination. Human placenta DNA (Sigma) was used as a control. The PCR programme was 94°C for 5 min, followed by 35 cycles at 94°C for 30 s, 58°C for 30 s, and 72°C for 30 s, with a final step of 72°C for 10 min. Cord blood and normal marrow were obtained with informed consent and approval from the South East Wales Research Ethics Committee and were performed in accordance with the ethical standards laid down in the 1964 Declaration of Helsinki. BM mononuclear cells were pooled from patients and CD34\ CD14\ and CD15^+^ cells purified using MiniMACS columns (Miltenyi Biotec., Surrey, UK). Cell purity was determined by flow cytometry (FACS Calibur) and data processed using the programme WinMDI2.8 (Purdue University Cytometry Labs). CD34+ cells were cultured in standard (RPMI-1640) medium supplemented with stem cell factor (SCF), interleukin (IL)-3, IL-6, granulocyte-macrophage colony-stimulating factor (GM-CSF), G-CSF, and FMS-like tyrosine ki-nase 3 ligand (FLT3). HeLa cells transfected with Cx43 (Martin et al. 2001) and HL-1 cells, a mouse atrial cardio-myocyte tumor cell line, were grown in Dulbecco's modified Eagle's medium (DMEM) or Clay combe medium (JRH Biosciences, Hertfordshire, UK), respectively, as described (Verma et al. 2008; White et al. 2004).

**Table 1:** PCR primers used to analyze 20 human connexins and N cadherin.

Connexins	Accession numbers	Forward & reverse primers	Product Size (bp)
Cx25	NM_198568	ggggacacacagaggaacat	192
		gctggttcaggcttaagtgg	
Cx26	NM_004004	cctgagtggggtcaacaagt	150
		gggacacagggaagaactca	
Cx30	NM_006783	tgcttaacgtggcagagttg	244
		ggttggtattgccttctgga	
Cx30.2	NM_181538	ggcactgggaattatcagga	250
		cgggacagattgcaggttat	
Cx30.3	NM_153212	tgaggatgggaactctgtcc	236
		gaccaaactacccccaacct	
Cx31	NM_024009	tctggcatggcttcaatatg	186
		tgtggcagatgaggtagcag	
Cx31.1	NM_005268	tccaagccctcagagaagaa	229
		ctgagcccagaaagatgagg	
Cx31.9	NM_152219	caagaggagttcgtgtgcaa	150
		gtgcatggagtagacgacga	
Cx32	NC_000023	tccctgcagctcatcctagt	156
		ccctg agatgtgg accttgt	
Cx36	NM_020660	ttcctagccctggacagaga	218
		gatgcagtgcgtagacctga	
CX37	BC027889	aagatctcggtggcagaaga	246
		tccaaccaccaacatg aag a	
Cx40	BC013313	taggcaaggtctggctcact	186
		tgatctgcagcacccagtag	
Cx40.1	NM_153368	aaagctctggttcgtcctca	229
		gtgcaggacatagacgctga	
Cx43	BC026329	atgagcagtctgcctttcgt	249
		tctgcttcaagtgcatgtcc	
Cx45	NM_005497	cacggtgaagcagacaagaa	217
		ttttcatgagcccatcttcc	
Cx46	NM_021945	catcttcaagacgctgttcg	168
		gccagcatgaagatgatgaa	
Cx47	NM_020435	gatccacaaccactccacct	168
		aaggcgtcatagcagacgtt	
Cx50	NM_005267	gagacactgccttcctacgc	175
		cgggggtctctactttctcc	
Cx59	NM_030772	aggtaacctcaagggccagt	205
		ccgcctaccaatgagattgt	
Cx62	NM_032602	ggcccagcagtgtatgattt	193
		tccgctatg ctg atccttct	
GAPDH	NM_002046	tgcatcctgcaccaccaact	329
		tgcctgcttcaccaccttc	
N-cadherin	NM_001792	acagtggccacctacaaagg	201
		ccgagatggggttgataatg	
			

Western blotting of cell proteins extracted at 4°C in sodium dodecyl sulfate with added proteolytic inhibitors (1 mg/ml leupeptin, 1 mg/ml aprotonin, and 0.5 mM phenylmethyl sulfonyl fluoride) was carried out in 4-12% *(w/v)* polyacrylamide gels. Separated proteins were electrophoretically transferred to nitrocellulose filters and nonspecific protein binding sites blocked before exposure to anti-connexin antibodies. After treatment with horseradish peroxide-conjugated secondary antibodies, signals were amplified using an enhanced chemiluminescence (ECL) solution (Amersham Biosci-ences, UK). Connexin antibodies were generated to a range of intracellular peptides linked to keyhole limpet haemocyanin (Oviedo-Orta et al. 2000) or were purchased from Zymed or Chemicon laboratories (USA). These antibodies bind to rodent and human connexins.

Coupling was measured by detection of dye transfer between cells. Monolayer cells were grown to confluence in 25-cm^2^ diameter flasks. Donor cells were loaded with 5 mM calcein (Molecular Probes), a fluorescent probe that permeates Cx37 and Cx43 gap junction channels (Veitch et al. 2004) and recipient cells with 5 μg/ml DiI C18 (Molecular Probes). After incubation in *5%* CO_2_ at 37°C for 30 min, the dye-loaded cells were washed with phosphate-buffered saline (pH7.4) and thenharvestedaftertreatment with trypsin. Cells were resuspended in culture medium and 2×10^5^ donor and recipient cells in a 1:1 ratio were cultured at 37°C in *5%* CO_2_ for 4 h. As controls, non-dye-loaded cells of each category were used. Dye transfer was evaluated by flow cytometry and repeated 3 to 4 times. Cells grown in suspension were treated as with confluent monolayers with omission of trypsin treatment. To study the involvement of gap junctional coupling, cells were treated for 30 min with the following gap junction inhibitors: 18α-glycyrrhetinic acid (18GA) or Gap 27 (sequence SRPTEKTIFII: residues 204–214 of Cx43) as stated in the figure legends. In some experiments, Gap 27 was substituted by a second Cx mimetic peptide Gap 26 (sequence VCYDKSFPISH-VR; residues 63–75 of Cx43) that, as previously shown (Evans and Leybaert 2007), also inhibits gap junctional communication.

## RESULTS

Adult BM and CB cells were fractionated into subpopula-tions of stated purity and RNA expression of 20 human connexins was examined by RT-PCR (Table 2). Cx37 expression was detected in bone marrow and cord blood CD34^+^ cells and in cord blood CD14^+^ monocyte cell populations. Cx43 was also detected in CB and BM derived CD34+ cells as well as in CB CD14+ cells. A signal was repeatedly observed with Cx26 (a connexin found in skin and the ear; Willecke et al. 2002) in CD14+ cells in CB but not in BM and is probably an artefact. Cx26 was not detected in CD34^+^ cells purified from cord blood or bone marrow. mRNA expression of N-cadherin, an adhesion protein expressed at low levels, provided a positive control in CD34^+^ cells from both sources. Freshly isolated CD34^+^ cells are a largely quiescent population; to determine whether the cell cycle status affected connexin expression, we repeated the analysis on CD34^+^ cells cultured in the presence of growth factors. Culturing of these cells for 13 days did not promote connexin mRNA expression.

**Table 2:** RT-PCR analysis of human connexin mRNA expression in progenitor stem cells.

	Human cord blood				Human bone marrow		
		
Cxs	CD34^+^ (93%)	CD14^+^ (95%)	CD15^+^ (92%)	CD34^+^ cultured for 10 days	CD34^+^ (86%)	CD14^+^ (80%)	CD15^+^ (98%)
Cx25	-	-	-	-	-	-	-
Cx26	-	+	-	-	-	-	-
Cx30	-	-	-	-	-	-	-
Cx30.2	-	-	-	-	-	-	-
Cx30.3	-	-	-	-	-	-	-
Cx31	-	-	-	-	-	-	-
Cx31.1	-	-	-	-	-	-	-
Cx31.9	-	-	-	-	-	-	-
Cx32	-	-	-	-	-	-	-
Cx36	-	-	-	-	-	-	-
Cx37	+	+	-	-	+	-	-
Cx40	-	-	-	-	-	-	-
Cx40.1	-	-	-	-	-	-	-
Cx43	+	+	-	+	+	-	-
Cx45	-	-	-	-	-	-	-
Cx46	-	-	-	-	-	-	-
Cx47	-	-	-	-	-	-	-
Cx50	-	-	-	-	-	-	-
Cx59	-	-	-	-	-	-	-
Cx62	-	-	-	-	-	-	-
N-Cad	+	-	-	-	+	-	-

*Note*. Numbers in parentheses indicate purity of subpopulation analyzed.

**Figure 1:**
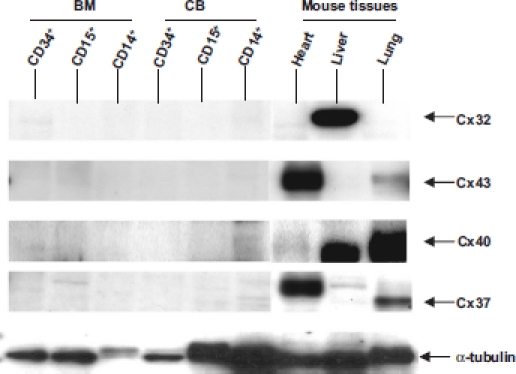
Analysis of Cx expression by CD34^+^, CD15^+^, and CD14^+^ bone marrow (BM) and cord blood (CB) cells and in mouse heart, liver, and lung by SDS-polyacrylamide gel electrophoresis. Mouse tissues were used as controls to verify that the antibodies were effective in staining Cx32, Cx43, Cx40, and Cx37. Protein addition to the lanes was monitored by staining the gels with tubulin antibodies. No connexins were detected in BM and CB cells.

Cx protein expression was examined by Western blotting. Since antibodies to the full range of Cxs are unavailable, we confined our attention to Cx32, Cx37, Cx40, and Cx43 using appropriate tissue controls expressing these connexins. Figure 1 shows that Cx32, Cx37, Cx40, and Cx43 could not be detected in BM and CB stem cell progenitor populations.

**Figure 2:**
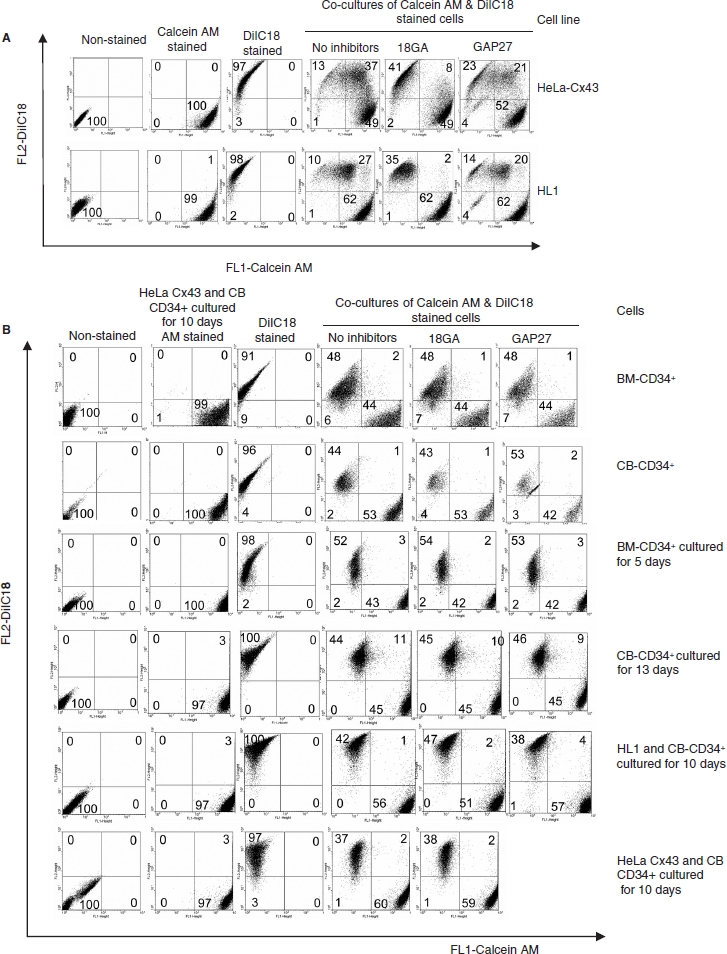
(**A**)Validation of dye transfer assay showing HeLaCx43 cells co-cultures with HL-1 cells and inhibition by 18GA (75 μM) and Gap 27 (150 μg/ml). (**B**) Transfer of calcein between BM or CB CD34^+^ cells cultured for 5, 10, or 13 days as well as in co-cultured with HL-1 and HeLa Cx43. The data represent a compromise between voltage and compensation settings.

We next examined whether CD34^+^ cells communicated via gap junctions with each other, as well as with other mammalian model cells expressing Cx43, by following intercellular transfer of calcein, a small fluorescent dye loaded into donor cells. To validate this assay of cell communication and the efficacy of the two gap junction inhibitors used, we demonstrated first that HeLa cells expressing Cx43 (previously shown to be communication competent on the basis of intracellular Ca transfer and electrical coupling; Paemeleire et al. 2000) transferred dye (Figure 2A). Also, HL-1 cells that display cardiac-type properties and express Cx43 and Cx37 (Verma et al. 2008) were coupled but to a lesser extent. In both instances, dye transfer was inhibited by the gap junction inhibitor 18GA and connexin mimetic peptide Gap 27. Dye transfer between freshly prepared BM or CB CD34+ was absent (Figure 2B).

**Figure 3:**
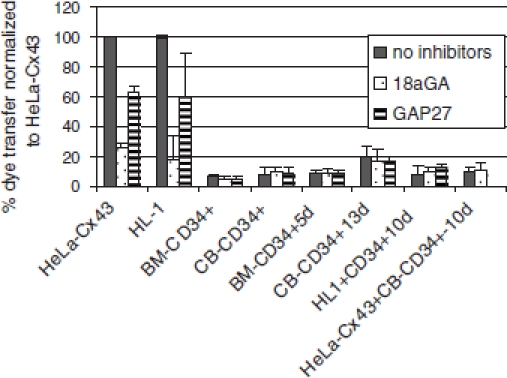
Bar chart summarizing the effects of gap junction inhibitors on dye transfer determined by fluorescence-activated cells sorting. Note that the low dye transfer detected in cells cultured for 5, 10, or 13 days was not inhibited by treatment with 18GA or Gap 27. Similar data showing the very low dye transfer between CD34^+^ cells was also obtained using Gap 26 (data not shown).

Low-level transfer was observed in cells that were cultured for 13 days. However, two peptide inhibitors of gap-junctional coupling had little effect on the low-level dye transfer detected, indicating that this was unlikely to be mediated by gap junctions. We also analyzed the capacity of the CD34^+^ cells to communicate across gap junctions by their coculture with HL-1 cells or with HeLa cells expressing Cx43 (Figure 2B). No dye coupling was observed between CD34^+^ cells and each of the Cx43-expressing cells used in these studies. The data are summarized in Figure 3.

## DISCUSSION

Several reports have claimed that adult human stem cells, especially autologous BM CD34^+^ subpopulations, administered directly into hearts of patients with ischemic damage, enhanced cardiac functioning (Balsam et al. 2004; Martin-Rendon et al. 2008; Murry et al. 2005; Psaltis et al. 2008; Roell et al. 2007; Segers and Lee 2008; Yip et al. 2008; Zhang et al. 2007). For such treatment to be effective, one likely scenario is that BM stem cells introduced into the heart become functionally integrated and are retained in cardiac tissue, possibly transdifferentiating into cardiac-like cells (Breitbach et al. 2007). Here, we addressed the thesis that key proteins enabling the functional integration of stem cells into heart may be connexins, especially Cx43, a protein that is expressed at high levels in myocardial tissue.

Connexins are proteins that oligomerize into dodeca-meric gap junction channels that facilitate direct intercellular cell communication, allowing metabolic, ionic, and electrical coordination of cell assemblies. Connexins are also present in the plasma membrane as unopposed hexameric hemichannels and these, when in open channel configuration, connect the cell interior to the external environments, allowing passage through the channels of, for example, ATP and calcium (Evans et al. 2006; Verma et al. 2008). Connexin hemichannels in the plasma membrane confer the independent property of cell-cell adhesion in addition to intercellular communication (Cotrina et al. 2008; Wong et al. 2006).

The present results show that CD34^+^ cells that display stem cell progenitor properties (Kawamoto et al. 2006) express very low levels of the 20 connexin iso-forms examined. Only Cx37 and Cx43 were detected at the mRNA level. Specifically, Cx32, present in primitive hematopoietic progenitor cells in mice (Hirabayashi et al. 2007), was not found. At the functional level, flow cytometry studies were carried out in the presence of specific inhibitors of gap junctions and these showed that CD34^+^ cells derived from adult BM and CB showed little capacity to communicate with each other, if at all, in a connexin-dependent manner; these cells also did not communicate with other cell types that express Cx43, including a cell line that has retained many cardiospecific properties. The use of two gap junction inhibitors, 18GA and the connexin mimetic peptides Gap 27 and Gap 26 (Evans and Leybaert 2007), reinforces the data. Taken together, therefore, the compositional and functional data suggest that gap-junctional communication occurring between the introduced adult progenitor stem cells and resident heart cells is unlikely. Since Cx43 and Cx37 enhance cell adhesion (Wong et al. 2006), the extremely low expression of these proteins also does not encourage any general hypothesis that connexins facilitate adhesive cell interactions between adult stem cells and cardiac cells. Other studies are in agreement with this conclusion. For example, myogenic cells grafted into infarcted myocardium failed to induce electromechanical coupling with heart cells (Leobon et al. 2003), and hematopoietic stem cells failed to transdiffer-entiate into cardiac myocytes in damaged regions of heart muscle (Gallo et al. 2007). BM-derived mesenchymal cells from rodents express some cardiacspecific markers, including Cx43, but did not differentiate into functioning cardiomyocytes (Rose et al. 2008).

The distribution of connexins and gap junctions in embryonic and adult stem cells has been reviewed (Huettner et al. 2006; Wong et al. 2008). Mouse embryonic stem cells, in contrast to results presently obtained with adult human stem cells, express a range of connexins, especially Cx31, Cx43, and Cx45 (Worsdorfer et al. 2008). However, adult epidermal stem cells do not communicate via gap junctions (Matic et al. 2002) and Cx43 has been shown to be a negative marker for progenitor stem cells in human limbal epithelium (Chen et al. 2006). These diverse data, taken together with the very low expression of Cx43 in adult bone marrow cells, do not support the attribution of major roles for Cx proteins in the integration of bone marrow CD34^+^ cells into heart tissue. Indeed, they reinforce a broad view that a lack of or a low connexin expression is a characteristic property of many adult stem cells. However, a possible role for Cx-mediated communication cannot be entirely ruled out because the extremely low levels of mRNA encoding Cx43 and Cx37 recorded may be sufficient to allow a few proteins to assemble into gap junction channels.

The conclusion that connexins are unlikely to be key players in improving the performance of hearts damaged by annoxia after infarction prompts a discussion of other possible mechanisms. Tunneling nanotubes extending from cell to cell (Koyanagi et al. 2008; Watkins and Salter 2005) may explain the very low level dye transfer that was not inhibited by the two gap junction inhibitors. BM cells may enhance endothelial cell function, possibly by influencing release of ATP and its breakdown products with Cx37 and/or Cx43 hemichannels (Eltzschig et al. 2006), pointing the finger at the possible importance of endothelial cells, i.e., the vasculature in cardiac repair. Recently, BM cell supernatants were shown to promote proangiogenetic effects, suggesting that paracrine factors, including inflammatory chemokines, cytokines and especially growth factors, e.g., fibroblast growth factor (FGF)-9 are key molecules released by BM cells that act on endothelial cells or myocytes in hearts damaged by hypoxia (Drexler and Wollert 2009). Finally, in attempts to identify optimum cell types to drive robust cardiac myogenesis, cardiac progenitor cells have been identified in embryonic/ adult hearts and these could also be key players in inducing cardiac repair (Nahrendorf et al. 2007).

In conclusion, the results show that connexin proteins, assembled into hemichannels and gap junctions in the plasma membrane where they underpin intercellular communication, are expressed at such low levels by progenitor CD34^+^ cells and myeloid lineage cell types that they are unlikely to provide a monocausal explanation for clinical data demonstrating beneficial outcomes of application of autologous adult BM stem cells in cardiac repair. Deeper knowledge of adult stem cell plasticity (Chen et al. 2006; Jones and Wagers 2008; Raff 2003) and a better understanding of the complex biochemical mechanisms underpinning cell differentiation and interactions, and especially paracrine factors released by BM cells, is required to unravel the variable clinical trial results.
